# Supplementary material for: Cultivation of Prevotella copri in a medium supplemented with tomato juice suppresses the bacteria-induced intestinal permeability in Caenorhabditis elegans
Source: PLoS One. 2025 Sep 5;20(9):e0331446. doi: 10.1371/journal.pone.0331446 (PMC12412950; doi:10.1371/journal.pone.0331446)
Supplement: Attachment — Submitted filename: Response to Reviewers.docx [file pone.0331446.s006.docx]

Response to Reviewers

We thank the academic editor and reviewers for the time and effort spent on the manuscript. The suggestions and comments have improved the quality of the manuscript. We have made responses to the comments point-by-point, and revised the manuscript accordingly, as follows. We hope our revisions fully meet your requirements.

Response to the academic editor

**Point 1:** Specifically, more explanation should be provided for the study design, including the use of 1% v/v tomato juice.

**Response:** Thank you very much for your comment. We chose to use commercially available tomato juice as the test sample because it provides more consistent quality compared to fresh tomato juice, which enhances reproducibility in future human intervention studies. Additionally, based on our previous research, we have found that supplementing the gut microbiota culture system with food ingredients at a concentration of 1% (v/v) is sufficient to induce changes in the composition of various bacterial species. At this concentration, the effect of the test ingredient can be evaluated without significantly disturbing the nutrient balance of the BG medium. For these reasons, we used tomato juice at a concentration of 1% (v/v) in this study. We have added this explanation to the revised manuscript.

**Details of corrections in “traced” manuscript:**

(Page 6, Lines 91-97 in the revised manuscript with track changes)

The sentences have been added as follows: “Commercially available tomato juice was selected as the test sample due to its consistent quality compared to fresh juice and its suitability for use in future human intervention studies. Additionally, based on our previous findings, we confirmed that adding food ingredients to the gut microbiota culture system at a concentration of 1% (v/v) is sufficient to alter the composition of various bacterial species. Furthermore, at a concentration of 1% (v/v), we believe the impact on bacteria can be assessed without disrupting the nutrient composition inherent to the BG medium. Thus, a 1% (v/v) concentration of tomato juice was chosen for this study.”

**Point 2:** Reviewer #1 further pointed out the need to examine the viable counts of the three bacterial species.

**Response:** Thank you very much for your comment. In response to Reviewer #1’s suggestion, we attempted a colony formation assay for *P. copri*, the primary focus of this study. However, no colonies were observed. Given that *P. copri* is an obligate anaerobe, we suspect that its exposure to atmospheric oxygen after application to the NGM plates compromised its ability to form colonies. Therefore, in this study, we assessed bacterial counts by measuring turbidity. Further details can be found in our response to Reviewer #1, Point 7.

**Point 3:** In addition, reviewer #2 commented the lack of clarity for the statistical analyses used.

**Response:** Thank you very much for your comment. As mentioned in our response to Reviewer #2, Point 4, the statistical methods used for each analysis are described in the *Materials and Methods* section and in the respective figure legends. However, we agree that the rationale for choosing specific statistical tests could be more clearly explained. In particular, when comparing continuous variables between groups, it would have been more appropriate to first assess the equality of variances before selecting the statistical method.

Therefore, we have revised the statistical analysis procedures as follows: For comparisons between two groups, an *F*-test was performed to assess the equality of variances. For comparisons among three groups, Bartlett’s test for homogeneity of variances was used. If a tendency toward unequal variances was observed (*P* < 0.1), Welch’s *t*-test was applied for both two- and three-group comparisons. In the case of three-group comparisons, *P*-values were adjusted using the Holm method. If no significant difference in variances was detected (*P* ≥ 0.1), indicating that the assumption of equal variances was met, Student’s *t*-test was used for two-group comparisons, and Dunnett’s test was used for three-group comparisons. We have updated the *Materials and Methods* section accordingly in the revised manuscript. Further details can be found in our response to Reviewer #2, Point 4.

Response to Reviewer #1

**Point 1:** In a previous epidemiological study, the authors investigated the relationship between human intestinal bacteria, tomato juice intake, and blood LPS-binding protein concentrations. As a result, they reported that there was a positive correlation between the population of intestinal bacteria three genera, Prevotella, Megamonas, and Streptococcus, and LPB, and a negative correlation between the intake of tomato juice and the amount of bacteria genus Streptococcus. Therefore, the authors used a model organism, the nematode C. elegans, to feed these bacteria cultured in a medium containing tomato juice and examined the intestinal permeability. The ideas in this study are considered novel and important. On the other hand, some controlled experiments and more careful consideration are needed to reach any conclusions. Additionally, the English text also needs further proofreading.

**Response:** Thank you very much for your valuable comments and constructive feedback. We have now conducted comprehensive English language proofreading of the entire manuscript to improve clarity and readability, and all corrections made during this process are retained as track changes in the Word file.

**Point 2:** The title and short title are unclear and should be corrected.

For example, “Cultivation of P. copri in a medium supplemented with tomato juice suppresses the bacteria-induced intestinal permeability in C. elegans.” or “P. copri cultured with tomato juice supplement abolishes bacteria-induced intestinal permeability in C. elegans.”

**Response:** Thank you very much for your comment. In accordance with your recommendation, we have revised both the manuscript title and the short title to improve clarity and better reflect the content of the study.

**Details of corrections in “traced” manuscript:**

(Page 1, Lines 1-4 in the revised manuscript with track changes)

The sentences have been corrected as follows: ~~Tomato juice supplemented cultivation of~~ *~~Prevotella copri~~* ~~inhibits intestinal permeability enhancing effect of the bacteria in~~ *~~Caenorhabditis~~* *~~elegans~~*Cultivation of *Prevotella copri* in a medium supplemented with tomato juice suppresses the bacteria-induced intestinal permeability in *Caenorhabditis elegans*

(Page 1, Lines 6-7 in the revised manuscript with track changes)

The sentences have been corrected as follows: ~~Tomato juice cultivation inhibits~~ *~~P~~*~~.~~ *~~copri~~*~~'s effect on nematode intestinal permeability~~Tomato juice-supplemented culture reduces *P*. *copri*-induced intestinal permeability in *C*. *elegans*

**Point 3:** To assert that the three bacteria used in this experiment increase the intestinal permeability of C. elegans, the results of the Smurf assay of E. coli OP-50 cultured for up to 8 days as a control are needed.

**Response:** Thank you very much for your comment. In response, we conducted the Smurf assay using *E. coli* OP50 and compared the proportion of Smurf individuals to those observed in worms fed with *Prevotella*, *Megamonas*, and *Streptococcus*. As a result, no significant differences were observed in the proportion of Smurf individuals between the *E. coli* OP50 group and any of the other bacterial groups.

*E. coli* OP50 is widely used as the standard laboratory diet for *C. elegans* due to its slow growth and the formation of a thin bacterial lawn on NGM agar, which facilitates microscopic observation. It is also favored for its safety and ease of handling. However, wild *C. elegans* are naturally associated with a diverse gut microbiota. Previous studies have shown that feeding worms a bacterial mixture resembling their natural microbiota can extend lifespan compared to worms fed with *E. coli* OP50 (Stover MA et al., *Front Physiol.* 2023 Sep 12;14:1207705). These findings suggest that *E. coli* OP50 may not be optimal for maintaining gut barrier function.

Therefore, in the present study, we did not place primary emphasis on comparisons with *E. coli* OP50. Instead, we focused on assessing the effects of culturing each bacterial strain in TJ medium and their individual influence on host intestinal permeability. We have added these experimental results and their interpretation to the revised manuscript.

**Details of corrections in “traced” manuscript:**

(Page 2, Lines 23-27 in the revised manuscript with track changes)

The underlined sentence has been added as follows: We cultured *Escherichia coli* OP50 (the standard *C*. *elegans* food), *Prevotella* (*P*.) *copri* JCM 13464^T^, *Megamonas funiformis* JCM 14723^T^, and *Streptococcus salivarius* JCM 5707^T^ in either normal medium or medium containing 1% (v/v) tomato juice (TJ medium), fed these bacteria to *C. elegans* for three days, and evaluated intestinal permeability using the Smurf assay.

(Page 7, Lines 115-117 in the revised manuscript with track changes)

The underlined sentence has been added as follows: The bacterial strains used in this study are summarized in Table 1. All strains, except for *E*. *coli* OP50, were obtained from the Microbe Division/Japan Collection of Microorganisms (JCM), RIKEN BioResource Research Center (Ibaraki, Japan).

(Page 22, Lines 369-378 in the revised manuscript with track changes)

The underlined sentence has been added as follows: *E. coli* OP50, *P. copri* JCM 13464^T^, *M. funiformis* JCM 14723^T^, and *S. salivarius* JCM 5707^T^ were grown anaerobically in BG or TJ medium, concentrated, and added to NGM. *C. elegans* were fed *E*. *coli* OP50, the standard diet, for 4 days after hatching, and then fed the test organisms applied to NGM from days 5 to 8. On day 8, intestinal permeability of *C. elegans* was evaluated using the Smurf assay (Fig 1A). For *P. copri* JCM 13464^T^, the proportion of Smurfs was significantly lower in *C. elegans* that had ingested bacteria grown on the TJ medium than in those grown on the BG medium (Fig 1B). For *E. coli*, *M. funiformis* and *S. salivarius*, no significant differences were observed between the two media. No significant difference in the proportion of Smurf individuals was observed between *E*. *coli* OP50 and *P*. *copri*, *M. funiformis,* or *S. salivarius* cultured on BG medium.

(Page 35, Lines 600-614 in the revised manuscript with track changes)

The sentences have been added as follows: As noted in the Introduction, *Prevotella*, *Megamonas*, and *Streptococcus* are gut bacterial genera implicated in increased intestinal permeability in human epidemiological studies. Therefore, we compared the intestinal permeability of *C*. *elegans* fed these bacteria with that of worms fed *E*. *coli* OP50, the standard laboratory diet for *C*. *elegans*. However, no significant differences were observed in the proportion of "Smurf" phenotype individuals between worms fed *E*. *coli* OP50 and those fed *P*. *copri* JCM 13464^T^, *M. funiformis* JCM 14723^T^, or *S. salivarius* JCM 5707^T^. *E*. *coli* OP50 is commonly used as the standard food source for *C*. *elegans* because it grows slowly and forms a thin bacterial lawn on NGM agar plates, facilitating microscopic observation of the worms. Moreover, it is well-suited for laboratory use due to its safety and ease of cultivation. Nonetheless, wild *C*. *elegans* harbor diverse gut microbiota. Previous studies have shown that feeding *C*. *elegans* a bacterial mixture resembling the natural gut microbiota of wild nematodes can extend lifespan compared to worms fed *E*. *coli* OP50 [28]. This suggests that *E*. *coli* OP50 may not be the optimal diet for maintaining gut barrier function. Therefore, this study does not focus on direct comparisons between *E*. *coli* OP50 and the other tested bacterial strains. Instead, we focused on the effects of culturing each bacterial species in TJ medium, emphasizing their individual impact on host intestinal permeability.

(Figures)

The results of the Smurf assay conducted on C. elegans fed with E. coli OP50 cultured in either BG or TJ medium have been added to Fig. 1B.

**Point 4:** In addition, the authors should conduct experiments in which these three types of bacteria are fed to L1 larval nematodes from day 1 to day 8. Whether the nematodes can digest these bacteria and grow normally is also important for understanding the physiological state of the nematodes in the experiment in which bacteria are added from day 4.

**Response:** Thank you very much for your comment. To assess the potential effects of the test bacterial strains on *C. elegans* development, we conducted experiments in which the strains were administered from the time of hatching (L1 stage). In control animals fed *E. coli* OP50 cultured on BG medium, body size increased steadily through day 5 post-hatching (S1 Table). In contrast, worms fed with *Prevotella*, *Megamonas*, or *Streptococcus* from hatching exhibited slower somatic growth. Moreover, *Prevotella*-fed worms showed a pronounced reduction in egg production (S2 Table).

Based on these findings, to minimize potential confounding effects of the test strains on worm development, we chose to begin bacterial administration on day 5 post-hatching, when somatic growth is largely complete. This experimental rationale and the relevant data have been added to the revised manuscript.

**Details of corrections in “traced” manuscript:**

(Pages 10-11, Lines 165-173 in the revised manuscript with track changes)

The sentences have been added as follows: To evaluate the potential effects of the test bacterial strains on *C. elegans* development, we examined whether administering these strains from the time of hatching would influence worm growth. In control animals fed *E. coli* OP50 cultured on BG medium, body size increased steadily through day 5 post-hatching (S1 Table). In contrast, worms fed *P. copri* JCM 13464^T^, *M. funiformis* JCM 14723^T^, or *S. salivarius* JCM 5707^T^ from hatching exhibited slower body growth. Furthermore, worms fed *P. copri* JCM 13464^T^ exhibited a marked reduction in egg production (S2 Table). Based on these findings, to minimize any confounding effects of the test strains on somatic development, we began administration of the bacteria on day 5 post-hatching, when body growth is considered to plateau. The final *C. elegans* feeding schedule was as follows:

(Page 51, Lines 960-967 in the revised manuscript with track changes)

The sentences have been added as follows:

**S1 Table. Body size of *Caenorhabditis elegans* on days 4–8 of adulthood, after exposure to test bacteria from hatching.** *Caenorhabditis elegans* were fed test bacterial strains cultured on either BG or TJ medium from the time of hatching until day 8 post-hatching. Body size was measured from day 4 to day 8 post-hatching.

**S2 Table. Brood size of *Caenorhabditis elegans* on days 4–8 of adulthood, after exposure to test bacteria from hatching.** *Caenorhabditis elegans* were fed test bacterial strains cultured on either BG or TJ medium from the time of hatching until day 8 post-hatching. Brood size was measured from day 4 to day 8 post-hatching.

(Tables)

The evaluation results of body size and brood size in C. elegans have been included in S1 and S2 Tables, respectively.

**Point 5:** Could the large indentations seen in SEM observations (Fig. 3) be debris of bacterial origin? Why would washing bacteria with sterile water produce indentations? An explanation is needed.

**Response:** Thank you very much for your comment. It has been reported that in *E. coli*, exposure to a hypotonic environment leads to an influx of water into the cytoplasm, which activates mechanosensitive channels (Hartmann M. et al., *Antimicrob Agents Chemother.* 2010 Aug;54(8):3132–42). This activation results in the release of water and ions into the periplasmic space, causing it to expand. The subsequent structural changes in the outer membrane may appear as surface undulations or localized indentations under SEM. Similar morphological features were observed in *P. copri* JCM 13464T following washing with sterile distilled water, as shown in Fig. 3. These observations suggest that the indentations are not bacterial debris but rather a physiological response to osmotic stress. We have included this explanation in the revised manuscript.

**Details of corrections in “traced” manuscript:**

(Pages 25-26, Lines 435-442 in the revised manuscript with track changes)

The sentences have been corrected as follows: It has been reported that in *E. coli*, exposure to a hypotonic environment causes an influx of water into the cytoplasm, which in turn activates mechanosensitive channels [25]. This leads to the release of water and ions into the periplasmic space, resulting in its expansion and causing the outer membrane to display surface undulations and localized indentations, which are similar to those seen in *P. copri* JCM 13464^T^ in Fig 3. ~~To assess the viability of~~ *~~P~~*~~.~~ *~~copri~~* ~~JCM 13464~~^~~T~~^ ~~after washing with sterile distilled water,~~ To investigate whether osmotic stress might affect *P. copri* JCM 13464^T^ viability under our experimental conditions, the bacteria were stained with propidium iodide (PI) and subjected to flow cytometry analysis after being left unwashed or washed once or twice with sterile distilled water.

**Point 6:** Furthermore, shouldn't saline be used in general to wash away bacteria? Osmotic pressure changes occur. Washing PI-positive cells with sterile water may cause the bacteria to burst and die due to osmotic stress, leading to a decrease in PI positivity and an increase in debris.

**Response:** Thank you very much for your comments. We agree with the reviewer’s comment that washing the bacterial cells with sterile distilled water likely caused osmotic stress, potentially leading to cellular damage. We also acknowledge that using physiological saline would have been more appropriate for maintaining cell integrity during washing. Nonetheless, we included the results obtained from the water-washed bacteria (Figs. 2–4) in the manuscript because they were informative in generating a useful hypothesis—that viable *P. copri* JCM 13464^T^ may contribute to increased intestinal permeability. Importantly, in the experiments shown in Figs. 5 and 6, bacterial cells were not washed with distilled water.

**Point 7:** The decrease in absorbance seen in Figure 6 may be due to the lysis of bacteria during storage. Furthermore, this lysis appears to be accelerated by culturing with TJ. In the washing with sterile water (Figures 3 and 4 exp) and the experiment in Figure 6, it is necessary to measure the number of live bacteria by measuring the number of colonies formed.

**Response:** Thank you very much for your comments. Among the points raised, we recognize that the colony assay of bacteria washed with distilled water (Figs. 3 and 4) may not be essential to the main conclusions of this manuscript. Therefore, we explored whether viable cell counts could be assessed using colony formation, in addition to turbidity measurements, for the experiments shown in Fig 6. To this end, we prepared BG agar plates by adding 1.5% (w/v) agar to the BG medium used in this study. One milliliter of *P. copri* JCM 13464^T^ culture, taken from a 10 mL BG liquid culture during subculture, was subjected to pour plating on BG agar and incubated at 37°C under anaerobic conditions for three days. This resulted in the formation of visible colonies (A in the figure below).

However, when *P. copri* JCM 13464^T^ was recovered from NGM plates—prepared and handled in the same manner as in Fig. 6—and 1 mL of the recovered suspension was plated on BG agar, no colonies were observed (B in the figure below). Since *Prevotella* is an obligate anaerobe and was exposed to atmospheric oxygen for four days after application onto the NGM surface, it is likely that the bacteria had lost their colony-forming ability.

Based on these results, we concluded that evaluating viable cells using colony formation was not feasible under the current experimental conditions. Therefore, we used changes in turbidity as an alternative means to assess bacterial lysis. We have added this information to the manuscript.

**Point 8:** One hypothesis is that when cultured with the addition of tomato juice, the bacteria produces antibiotics, but this hypothesis is difficult to understand because antibiotic-producing bacteria generally have mechanisms for conferring resistance to the antibiotics themselves.

**Response:** Thank you very much for your comments. As the reviewer rightly pointed out, it is indeed reasonable to assume that *P. copri* JCM 13464^T^ possesses resistance to any antimicrobial substances it may produce. Therefore, to better understand the impact of TJ and L-(-)-3-phenyllactic acid (L-(-)-3-PLA) on the survival of *P. copri* JCM 13464^T^, we decided to culture *P. copri* JCM 13464^T^ in BG medium supplemented with L-(-)-3-PLA and then assess its survival rate after application to NGM plates.

We quantified L-(-)-3-PLA concentrations in culture supernatants. Calibration curves using pure L-(-)-3-PLA were generated to calculate concentrations based on peak areas obtained by LC-MS/MS analysis. The results indicated that the concentration of L-(-)-3-PLA in the TJ medium culture supernatant of *P. copri* JCM 13464T was 5.5 μM.

Subsequently, we added 5.5 μM of L-(-)-3-PLA to BG medium to match the concentration detected in the *P. copri* JCM 13464T TJ culture supernatant. After culturing test bacterial strains in this medium, we assessed their survival rates following application to NGM plates. The addition of L-(-)-3-PLA to the BG medium inhibited the growth of *S. salivarius* JCM 5707^T^ but did not affect the growth or survival of *P. copri* JCM 13464^T^ and *M. funiformis* JCM 14723^T^. This suggests that L-(-)-3-PLA alone does not replicate the effects observed in TJ medium.

Although the resistance mechanism of *Prevotella* to L-(-)-3-PLA has not been elucidated, the unaffected survival of *P. copri* JCM 13464^T^—which produces PLA—even under L-(-)-3-PLA supplementation, and the inhibited growth of *S. salivarius* JCM 5707^T^—which does not produce PLA—suggest that *Prevotella* may possess a resistance mechanism against L-(-)-3-PLA. It has been reported that the activity of superoxide dismutase increases in *Staphylococcus aureus* cultured in the presence of PLA (Jiang YH et al., *J Dairy Sci.* 2022 Nov;105(12):9463–9475). Additionally, when the antioxidant activity gene *soxS* in *E. coli* is activated by genetic modification, the growth of *E. coli* in PLA-supplemented medium significantly increases (Zhou XL et al., *Adv Biotechnol (Singap).* 2024 Apr 2;2(2):15). Therefore, based on these previous reports, one of the mechanisms underlying the antibacterial action of L-(-)-3-PLA may involve the induction of oxidative stress, and enhancing antioxidant activity could be a mechanism of resistance to L-(-)-3-PLA.

In this study, LC-MS/MS analysis showed a decrease in the levels of 2,3,4,9-Tetrahydro-1H-β-carboline-3-carboxylic acid and Cyclo(phenylalanyl-prolyl) in *P. copri* JCM 13464^T^ cells when cultured in TJ medium. These compounds have been reported to possess radical scavenging ability (Ichikawa M et al., *Biofactors.* 2002;16(3-4):57–72; Bojarska L et al., *Biomolecules.* 2021 Oct 14;11(10):1515). These findings suggest that *P. copri* JCM 13464^T^ may utilize these antioxidant components as part of its resistance mechanism to L-(-)-3-PLA. Culturing in TJ medium may have simultaneously increased L-(-)-3-PLA production and decreased antioxidant compound levels, thereby enhancing susceptibility to L-(-)-3-PLA. We have added this information to the manuscript.

**Details of corrections in “traced” manuscript:**

(Page 3, Lines 39-43 in the revised manuscript with track changes)

The sentences have been added as follows: In contrast, the levels of known antioxidants such as 2,3,4,9-tetrahydro-1H-β-carboline-3-carboxylic acid and Cyclo(phenylalanyl-prolyl) were decreased. Culturing *P. copri* in normal medium supplemented with L-(-)-3-PLA alone did not reduce survival, suggesting that both L-(-)-3-PLA accumulation and the depletion of antioxidants contribute to reduced viability.

(Pages 8-9, Lines 131-135 in the revised manuscript with track changes)

The sentences have been corrected as follows: Specifically, 100 mL of BG medium, BG medium supplemented with 1% (v/v) commercial 100% tomato juice (KAGOME CO., LTD., no salt added, sterilized at 121°C for 15 minutes) (TJ medium), or BG medium supplemented with 5.5 μM of L-(-)-3-PLA was inoculated with 1% (v/v) of each bacterial culture and incubated overnight at 37°C.

(Pages 18-19, Lines 311-315 in the revised manuscript with track changes)

The sentences have been added as follows: Standard curves for quantifying the concentration of L-(-)-3-PLA in culture supernatants were prepared by dissolving L-(-)-3-PLA (Tokyo Chemical Industry Co., Ltd., Tokyo, Japan) in methanol at concentrations of 1, 5, 10, 50, and 100 μM. Similarly, standard curves for bacterial samples were prepared by dissolving L-(-)-3-PLA in methanol at concentrations of 0.00064, 0.0032, 0.016, 0.08, 0.4, 2, 10, and 50 μM. These samples were also filtered and transferred to HPLC vials.

(Page 20, Line 341 in the revised manuscript with track changes)

The sentences have been added as follows: The concentration of L-(-)-3-PLA in each sample was calculated based on the standard curve.

(Page 30 , Lines 511-519 in the revised manuscript with track changes)

The sentences have been added as follows: (C) The experiment was conducted as described for (B), with the following modifications. In addition to *Prevotella copri* JCM 13464^T^, *Escherichia coli* OP50, *Megamonas funiformis* JCM 14723^T^, and *Streptococcus salivarius* JCM 5707^T^ were also tested. The bacteria were cultured overnight in BG or TJ medium, with a group receiving BG medium supplemented with 5.5 μM of L-(-)-3-phenyllactic acid (L-(-)-3-PLA). After centrifugation and application to NGM, the bacteria were stored at 20°C for 4 days. Following resuspension in BG medium and adjustment of turbidity, the cultures were anaerobically incubated at 37°C for 2 days. Absorbance at 660 nm was measured from the first day (before the start of incubation) to the second day. Data are presented as the change in absorbance at 660 nm from day 1 to day 2.

(Pages 32-33 , Lines 562-575 in the revised manuscript with track changes)

The sentences have been added as follows:

## The effects of L-(-)-3-PLA on the vulnerability of *P. copri* JCM 13464^T^ and intestinal permeability in *C. elegans*

To evaluate the impact of L-(-)-3-PLA on the vulnerability of *P. copri* JCM 13464^T^ and its effect on intestinal permeability in *C. elegans*, the concentration of L-(-)-3-PLA in the TJ culture supernatant of *P. copri* JCM 13464^T^ and in the bacterial suspension applied to NGM was calculated. The results showed that the concentration of L-(-)-3-PLA in the TJ culture supernatant of *P. copri* JCM 13464^T^ was 5.5 μM, whereas the concentration of L-(-)-3-PLA in the TJ medium-cultured *P. copri* JCM 13464^T^ bacterial suspension applied to NGM was 10 nM (0.5 pmol per 50 μL).

To evaluate the impact of L-(-)-3-PLA on the vulnerability of the bacterial strains, each strain was cultured in BG medium with or without supplementation of 5.5 μM L-(-)-3-PLA. The bacterial survival rate following application to NGM was then assessed. *S. salivarius* JCM 5707^T^ exhibited a significantly smaller change in turbidity in the BG medium supplemented with L-(-)-3-PLA compared to the control group cultured in BG medium alone. No significant differences in turbidity changes between groups were observed for *P. copri* JCM 13464^T^, *M. funiformis* JCM 14723^T^, and *E. coli* (Fig. 6C).

(Pages 38-39 , Lines 660-681 in the revised manuscript with track changes)

The sentences have been added as follows: Therefore, we added L-(-)-3-PLA at the same concentration (5.5 μM) detected in the TJ culture supernatant of *P. copri* JCM 13464^T^ to BG medium and cultured the bacterial strains. After applying them to NGM, we evaluated their post-application viability. The addition of L-(-)-3-PLA to BG medium inhibited the growth of *S. salivarius* JCM 5707^T^ after NGM application but had no observable impact on the growth or survival of *P. copri* JCM 13464^T^ and *M. funiformis* JCM 14723^T^. In other words, L-(-)-3-PLA alone did not exhibit the same modulatory as TJ medium. Although the resistance mechanism of *Prevotella* to L-(-)-3-PLA has not been elucidated, the unchanged survival of *P. copri* JCM 13464^T^ which produces PLA, under L-(-)-3-PLA supplementation, and the inhibited growth of *S. salivarius* JCM 5707^T^, which does not produce PLA (as described in the section below), suggest that *Prevotella* may possess a specific resistance mechanism against L-(-)-3-PLA. It has been reported that the activity of superoxide dismutase increases in *Staphylococcus aureus* cultured in the presence of PLA [36]. Additionally, when the antioxidant activity gene *soxS* in *E. coli* is activated by genetic modification, the growth of *E. coli* in PLA-supplemented medium is significantly enhanced [37]. Therefore, based on these previous reports and as noted above, one of the mechanisms of the antibacterial action of PLA involves the induction of oxidative stress, and enhancing antioxidant activity could serve as a mechanism of resistance to PLA. In this study, LC-MS/MS data showed a decrease in the levels of 2,3,4,9-Tetrahydro-1H-β-carboline-3-carboxylic acid and Cyclo(phenylalanyl-prolyl) in *P. copri* JCM 13464^T^ cells cultured in TJ medium, and these components have been reported to possess free radical-scavenging ability [38,39]. Therefore, it is plausible that *P*. copri JCM 13464^T^ may utilize these antioxidant components as part of its resistance mechanism to L-(-)-3-PLA. Additionally, culturing in TJ medium might have led to an increase in L-(-)-3-PLA production and a reduction in antioxidant compound levels, which could have contributed to enhanced susceptibility to L-(-)-3-PLA.

(Pages 51-52, Lines 970-972 in the revised manuscript with track changes)

The sentences have been corrected as follows: S4 Table. Effect of culturing in a BG medium supplemented with tomato juice or L-(-)-3-phenyllactic acid on the growth or survival rate of the tested bacterial strains.

(Figures)

The results of the viability assessment after culturing each test bacterial strain in BG medium containing L-(-)-3-PLA, applying them to NGM, and subsequently resuspending them in BG medium were added to Fig. 6C.

(Tables)

The results of Fig. 6C have been included in S4 Table.

**Point 9:** It remains to be confirmed whether heat treatment of the other two bacteria also alters the intestinal permeability of the nematode. This finding may simply be the result of P.copri bacteria being more easily lysed (dead) when cultured in a medium containing tomato juice and washed with sterile water. Examining the viable counts of the three bacteria after each treatment may provide the key to solving this mystery.

**Response:** Thank you very much for your comments. We understand your comment to refer to whether the effect of TJ medium cultivation on intestinal permeability is related to the susceptibility of each bacterial strain to lysis after application to NGM. As stated in our response to Point 7, *P. copri* JCM 13464^T^, being an obligate anaerobe, does not form colonies in a colony assay after NGM application. Therefore, in the additional experiments conducted to address this point, bacterial counts were assessed using turbidity measurements.

As with *P. copri* JCM 13464^T^ (Fig. 6B), *E. coli* OP50, *M. funiformis* JCM 14723^T^, and *S. salivarius* JCM 5707^T^ were cultured in BG or TJ medium, applied to NGM, and stored at 20°C under atmospheric conditions for four days. The bacteria were then resuspended in BG medium, and changes in turbidity were evaluated. As a result, *M. funiformis* JCM 14723^T^ showed a decrease in turbidity in both the BG and TJ medium groups, suggesting bacterial lysis; however, no significant difference was observed between the two groups. For *S. salivarius* JCM 5707^T^, a lower turbidity was observed in the TJ medium group compared to the BG medium group, although turbidity increased in both groups, suggesting bacterial growth. Therefore, the reduced turbidity in the TJ group for *Streptococcus* was interpreted as growth inhibition rather than enhanced lysis.

Based on these results, we concluded that the reduction in intestinal permeability induction observed only in *P. copri* JCM 13464^T^ after cultivation in TJ medium is likely related to increased susceptibility to lysis after application to NGM. We have added this information to the manuscript.

**Details of corrections in “traced” manuscript:**

(Page 2, Lines 33-35 in the revised manuscript with track changes)

The sentences have been corrected as follows: Furthermore, *P. copri* grown in TJ medium exhibited a lower survival rate after seeding on nematode growth medium, an effect not observed in other strains.

(Page 16, Lines 275 in the revised manuscript with track changes)

The sentences have been corrected as follows: Growth and survival assay of bacteria

(Page 17, Lines 282-284 in the revised manuscript with track changes)

The sentences have been corrected as follows: In the post-NGM viability test, NGM plates seeded with *P. copri* JCM 13464^T^, *M. funiformis* JCM 14723^T^, *S. salivarius* JCM 5707^T^, or *E. coli* OP50-were opened on a clean bench, 2 mL of BG medium was added, and the pellets were gently tapped with a Conradi rod to suspend the bacteria.

(Pages 28-29, Lines 491-501 in the revised manuscript with track changes)

The sentences have been added as follows: As shown in Fig. 1A, the effect of bacterial cultivation in TJ medium on intestinal permeability varied among the bacterial strains, with only *P*. *copri* JCM 13464^T^ showing a decrease in the proportion of Smurf individuals among the four tested strains. We assessed whether this phenomenon was related to the bacterial survival rate following NGM application. The method was the same as that in Fig. 6B; however, for the culture duration after recovery of the bacteria from NGM, the turbidity change was measured until the following day, as in the actual *C. elegans* cultivation, where new plates are used after one day. As a result, *M. funiformis* JCM 14723^T^ showed a decrease in turbidity in both BG and TJ medium groups, but no significant difference between groups was observed (Fig. 6C, S4 Table). Both *S. salivarius* JCM 5707^T^ and *E. coli* OP50 exhibited an increase in turbidity in both BG and TJ medium groups. For *S. salivarius* JCM 5707^T^, the increase in turbidity was suppressed in the TJ medium group compared to the BG medium group, while no significant difference was observed between BG and TJ medium groups for *E. coli* OP50.

(Page 29, Line 503-504 in the revised manuscript with track changes)

The sentences have been corrected as follows: Fig 6. Effect of culturing in BG medium supplemented with tomato juice or L-(-)-3-phenyllactic acid on the growth or survival rate of the tested bacterial strains.

(Page 30 , Lines 511-519 in the revised manuscript with track changes)

The sentences have been added as follows: (C) The experiment was conducted as described for (B), with the following modifications. In addition to *Prevotella copri* JCM 13464^T^, *Escherichia coli* OP50, *Megamonas funiformis* JCM 14723^T^, and *Streptococcus salivarius* JCM 5707^T^ were also tested. The bacteria were cultured overnight in BG or TJ medium, with a group receiving BG medium supplemented with 5.5 μM of L-(-)-3-phenyllactic acid (L-(-)-3-PLA). After centrifugation and application to NGM, the bacteria were stored at 20°C for 4 days. Following resuspension in BG medium and adjustment of turbidity, the cultures were anaerobically incubated at 37°C for 2 days. Absorbance at 660 nm was measured from the first day (before the start of incubation) to the second day. Data are presented as the change in absorbance at 660 nm from day 1 to day 2.

(Page 36, Lines 618-630 in the revised manuscript with track changes)

The sentences have been added as follows: As part of our investigating into the underlying mechanism, we compared the survival rates of each bacterial strain following NGM application. The results showed that a decrease in turbidity, indicative of bacterial lysis, was observed only for *P. copri* JCM 13464^T^ and *M. funiformis* JCM 14723^T^. However, unlike *P. copri* JCM 13464^T^, *M. funiformis* JCM 14723^T^ did not exhibit any significant difference in the extent of lysis between the BG and TJ medium groups. For *S. salivarius* JCM 5707^T^, a decrease in turbidity was observed in the TJ medium group compared to the BG medium group; however, turbidity increased in both groups from day 1, indicating bacterial growth. Therefore, the lower turbidity in the TJ medium group for *S. salivarius* JCM 5707^T^ was considered to reflect growth inhibition rather than enhanced lysis. Furthermore, in the NGM application of this study, the plates were replaced daily. Therefore, it is assumed that the growth inhibition of *S. salivarius* JCM 5707^T^ by TJ medium does not affect the interpretation of Fig 1. These results suggest that the reduction in intestinal permeability induction by cultivation in TJ medium observed only in *P. copri* JCM 13464^T^ is associated with the ease of bacterial lysis following NGM application.

(Pages 51-52, Lines 970-972 in the revised manuscript with track changes)

The sentences have been corrected as follows: S4 Table. Effect of culturing in a BG medium supplemented with tomato juice or L-(-)-3-phenyllactic acid on the growth or survival rate of the tested bacterial strains.

(Figures)

The results of the viability assessment after culturing each test bacterial strain in TJ medium, applying them to NGM, and subsequently resuspending them in BG medium were added to Fig. 6C.

(Tables)

The results of Fig. 6C have been included in S4 Table.

Response to Reviewer #2

**Point 1:** In this study, the authors address a novel aspect of how tomato consumption may influence intestinal permeability through its effect on specific gut bacteria, which is relevant given the increasing interest in diet-microbiota interactions. However, the following comments and suggestions might be useful for improving the manuscript.

**Response:** Thank you very much for your thoughtful overall assessment of our study. We appreciate your recognition of the novelty and relevance of our work, and we will carefully consider your comments and suggestions to improve the manuscript.

**Point 2:** Utilizing C. elegans as a model organism for studying the impact of bacterial composition on intestinal permeability provides an efficient system for preliminary screening before moving to more complex models. However，the authors note that extrapolation to humans remains a challenge, it would be beneficial to discuss potential pathways or mechanisms that could be conserved across species, thereby justifying further studies in more complex models or human populations.

**Response:** Thank you very much for your comments. In humans, a higher prevalence of mucin-degrading bacteria in the intestine has been suggested to contribute to the disruption of intestinal barrier function (Png CW et al., *Am J Gastroenterol.* 2010 Nov;105(11):2420–8). *P. copri* is reported to possess genes encoding mucin-degrading enzymes, including GH20 (β-hexosaminidase), which cleaves N-acetylglucosamine/N-acetylgalactosamine (Glover JS et al., *Sci Rep.* 2022 May 19;12(1):8456). Therefore, it is hypothesized that one mechanism by which *P. copri* induces increased intestinal permeability involves disruption of the intestinal barrier via its mucin-degrading enzymes.

In *C. elegans*, the expression of mucin-type N-acetylgalactosamine transferases in the intestinal tract has been reported (Hagen F et al., *Trends in Glycoscience and Glycotechnology.* 2001;13(73):463–79), and mucin-like substances composed of N-acetylgalactosamine have been detected in *C. elegans* extracts (Staudacher E, *Molecules.* 2015 Jun 9;20(6):10622–40). These findings suggest the presence of N-acetylgalactosamine-containing mucin-like substances in the *C. elegans* intestine. Thus, it is considered that these mucin-like substances may serve as substrates for GH20 produced by *P. copri*.

From this perspective, *C. elegans* may serve as a useful model for studying mucin degradation-mediated increases in intestinal permeability, thereby mimicking certain aspects of human intestinal physiology. Supporting this view, *Pseudomonas aeruginosa*, which possesses a wide array of mucin-degrading enzymes (Aristoteli LP et al., *Infect Immun.* 2003 Oct;71(10):5565–75), has been implicated in the disruption of the human intestinal barrier leading to infection (Zaborina O et al., *Ann Clin Microbiol Antimicrob.* 2006 Jun 8;5:14). Furthermore, *P. aeruginosa* has been reported to degrade mucin (Hoffman CL et al., *mBio.* 2020 Mar 3;11(2):e00060–20) and increase intestinal permeability in *C. elegans* (Mohanapriya R et al., *The Microbe.* 2024 Sep;4:100155).

These pieces of information have been added to the revised manuscript. Additionally, since there were several scattered references to the potential for extrapolation to humans, we have now organized and clarified these statements for coherence and emphasis.

**Details of corrections in “traced” manuscript:**

(Pages 40, Lines 696-702 in the revised manuscript with track changes)

The following sentences have been moved to Pages 43-44, Lines 757-764: oxygen levels in the intestinal tract are extremely low [35], and there is concern that the oxidative stress to which intestinal bacteria are exposed is low. However, it has been suggested that in mice, intestinal D-amino acid oxidase metabolizes D-amino acids derived from intestinal bacteria to generate hydrogen peroxide, which in turn reduces the abundance of Bacteroidales (a superclass of *Prevotella*) in the intestinal tract [36]. Thus, it is possible that intestinal bacteria are affected by oxidative stress in the intestinal tract, and the decrease in *Prevotella* observed in this study may be replicated in the intestinal tract.

(Pages 42-44, Lines 738-769 in the revised manuscript with track changes)

The sentences have been added as follows: Finally, we discuss whether the results of this study can be replicated in humans. First, we discuss the suitability of this model for evaluating intestinal permeability. In humans, a higher prevalence of mucin-degrading bacteria in the intestine has been suggested to contribute to the disruption of intestinal barrier function [45]. *P. copri* is reported to possess genes encoding mucin-degrading enzymes, including GH20 (β-hexosaminidase), which cleaves N-acetylglucosamine/N-acetylgalactosamine [46]. Therefore, it is hypothesized that one of the mechanisms by which *P. copri* induces intestinal permeability involves the disruption of the intestinal barrier through mucin-degrading enzymes. The expression of mucin-type N-acetylgalactosamine transferases in the intestinal tract of *C. elegans* has been reported [47], and mucin-like substances composed of N-acetylgalactosamine have been detected in the *C. elegans* extracts [48], suggesting the presence of N-acetylgalactosamine-containing mucin-like substances in the *C. elegans* intestine. Therefore, it is considered that the mucin-like substances in *C. elegans* may serve as substrates for GH20, which is present in *P. copri*. From the perspective of increased intestinal permeability mediated by the degradation of intestinal mucin by *P, copri*, *C. elegans* may be considered a suitable organism that mimics human intestinal function. In fact, *Pseudomonas aeruginosa*, which possesses a potent array of mucin-degrading enzymes [49], has been implicated in the disruption of the intestinal barrier in humans, leading to infection [50]. Moreover, *Pseudomonas aeruginosa* has been reported to degrade mucin [51] and increase intestinal permeability in *C. elegans* [52]. Next, we discuss whether the effects of culturing *P. copri* in TJ medium observed in this study can be replicated in the human intestine. As mentioned above, the combination of L-(-)-3-PLA and oxidative stress has been suggested to induce bacterial lysis in *P. copri* Oxygen levels in the intestinal tract are extremely low [53], and there is concern that the oxidative stress experienced by intestinal bacteria is minimal. However, it has been suggested that in mice, intestinal D-amino acid oxidase metabolizes D-amino acids derived from intestinal bacteria to generate hydrogen peroxide, which in turn reduces the abundance of Bacteroidales (a superclass of *Prevotella*) in the intestinal tract [54]. Thus, it is possible that intestinal bacteria are affected by oxidative stress in the intestinal tract, and the decrease in *Prevotella* observed in this study may also be replicated in the intestinal tract. As mentioned above, based on the appropriateness of the evaluation model and the proposed mechanism of action of TJ on *P. copri*, it is hoped that the phenomena observed in this study may ultimately be replicated in humans. However, this study was conducted using *C. elegans* as a model organism and single bacterial strains *in vitro*, without considering host digestive processes and bacterial interactions in the gut, so extrapolation of these findings to humans remains a challenge for future research.

(Page 44, Lines 773-775 in the revised manuscript with track changes)

The following sentences have been moved to Pages 44, Lines 766-769: Because this study was conducted using *C. elegans* as a model organism and single bacterial strains in vitro, without considering host digestive processes and bacterial interactions in the gut, the extrapolation of these findings to humans remains a challenge for future research.

**Point 3:** Although L-(-)-3-phenyllactic acid accumulation is identified as a possible factor, more detailed investigation into how this compound specifically affects intestinal permeability and whether it directly causes increased vulnerability of P. copri would enhance the study's explanatory power.

**Response:** Thank you very much for your comments. In response to the reviewer’s suggestion, we conducted additional experiments to clarify the direct effects of L-(-)-3-PLA on intestinal permeability in *C. elegans* and the vulnerability of *P. copri* JCM 13464^T^.

First, we quantified L-(-)-3-PLA concentrations in both bacterial cell samples and culture supernatants. Calibration curves were established using pure L-(-)-3-PLA to calculate concentrations from the peak areas obtained via LC-MS/MS analysis. The results showed that the concentration of L-(-)-3-PLA in the culture supernatant of *P. copri* JCM 13464^T^ grown in TJ medium was 5.5 μM, while the concentration in the bacterial suspension applied to NGM was 10 nM.

Based on these findings, and as an additional experiment prompted by the reviewer’s comment, we added 5.5 μM of L-(-)-3-PLA to BG medium to match the concentration observed in the TJ culture supernatant. After culturing the test bacterial strains in this L-(-)-3-PLA-supplemented medium, we evaluated their survival after application to NGM plates. The addition of L-(-)-3-PLA inhibited the growth of *S. salivarius* JCM 5707^T^, but did not affect the growth or survival of *P. copri* JCM 13464^T^ and *M. funiformis* JCM 14723^T^. These results suggest that L-(-)-3-PLA alone does not fully replicate the effects observed in TJ medium.

Although the resistance mechanism of *Prevotella* to L-(-)-3-PLA is not fully understood, the unaffected survival of *P. copri* JCM 13464^T^—which produces PLA—even under L-(-)-3-PLA supplementation, and the inhibited growth of *S. salivarius*, which does not produce PLA, suggest that *Prevotella* may possess a specific resistance mechanism. *Staphylococcus aureus* has been reported to increase superoxide dismutase (SOD) activity when cultured in the presence of PLA (Jiang YH et al., *J Dairy Sci.* 2022), and activation of the antioxidant gene *soxS* in *E. coli* enhances its growth in PLA-supplemented medium (Zhou XL et al., *Adv Biotechnol (Singap).* 2024). These findings suggest that oxidative stress is a key mechanism of PLA’s antibacterial action, and increased antioxidant capacity may contribute to bacterial resistance. Our LC-MS/MS analysis further showed a decrease in the levels of 2,3,4,9-Tetrahydro-1H-β-carboline-3-carboxylic acid and Cyclo(phenylalanyl-prolyl) in *P. copri* JCM 13464^T^ cells cultured in TJ medium. These compounds have been reported to exhibit radical scavenging activity (Ichikawa M et al., *Biofactors.* 2002; Bojarska L et al., *Biomolecules.* 2021), suggesting that *P. copri* may utilize these antioxidant components in its resistance to L-(-)-3-PLA. Culturing in TJ medium may therefore simultaneously enhance L-(-)-3-PLA production while reducing antioxidant compound levels, leading to increased susceptibility to L-(-)-3-PLA.

Furthermore, based on the reviewer’s suggestion to examine the direct effects of L-(-)-3-PLA on intestinal permeability, we conducted another additional experiment in which *C. elegans* were fed with NGM plates supplemented with L-(-)-3-PLA. Previous studies have shown that PLA suppresses intestinal permeability in mice (Kim D et al., *Eur J Pharmacol.* 2024) and in human intestinal epithelial cell lines (Wei L et al., *Appl Environ Microbiol.* 2025). While its effects in *C. elegans* had not previously been reported, PLA feeding has been shown to extend lifespan via SKN-1/AFTS-1 activation (Kim J et al., *Nat Commun.* 2024), and SKN-1 activation has been associated with the maintenance of intestinal barrier function (Zhao Y et al., *Nanotoxicology.* 2016). In our experiment, feeding *C. elegans* with 10 nM L-(-)-3-PLA—the same concentration found in the *P. copri* TJ-cultured sample—significantly suppressed intestinal permeability compared to the untreated group.

Taken together, these additional findings suggest that the suppression of intestinal permeability observed in worms fed with *P. copri* JCM 13464^T^ cultured in TJ medium may involve both: (1) bacterial lysis, and (2) the direct barrier-strengthening effect of L-(-)-3-PLA derived from *P. copri*. We have added these findings to the revised manuscript.

**Details of corrections in “traced” manuscript:**

(Pages 2-3, Lines 35-45 in the revised manuscript with track changes)

The sentences have been corrected as follows: Liquid chromatography-tandem mass spectrometry analysis revealed that *P. copri* cultured in TJ medium accumulated L-(-)-3-phenyllactic acid (L-(-)-3-PLA), a compound known for its antibacterial properties through oxidative stress and its protective effects on the intestinal barrier. In contrast, the levels of known antioxidants such as 2,3,4,9-tetrahydro-1H-β-carboline-3-carboxylic acid and Cyclo(phenylalanyl-prolyl) were decreased. Culturing *P. copri* in normal medium supplemented with L-(-)-3-PLA alone did not reduce survival, suggesting that both L-(-)-3-PLA accumulation and the depletion of antioxidants contribute to reduced viability. Additionally, L-(-)-3-PLA directly suppressed intestinal permeability in *C. elegans*. In conclusion, the results of this study suggest that TJ may inhibit increased intestinal permeability through both rendering *P. copri* vulnerable and the direct effects of L-(-)-3-PLA.

(Pages 8-9, Lines 131-135 in the revised manuscript with track changes)

The sentences have been corrected as follows: Specifically, 100 mL of BG medium, BG medium supplemented with 1% (v/v) commercial 100% tomato juice (KAGOME CO., LTD., no salt added, sterilized at 121°C for 15 minutes) (TJ medium), or BG medium supplemented with 5.5 μM of L-(-)-3-PLA was inoculated with 1% (v/v) of each bacterial culture and incubated overnight at 37°C.

(Page 10, Lines 158-162 in the revised manuscript with track changes)

The sentences have been added as follows: NGM containing L-(-)-3-PLA was prepared according to previously reported methods [16]. Specifically, L-(-)-3-PLA was added to the NGM to achieve final concentrations of 10 nM or 100 nM during the cooling phase, after autoclaving and before solidification. After the NGM had cooled and solidified, 50 μL of the cultured and concentrated *E. coli* OP50 suspension, as described above, was applied to the agar.

(Pages 18-19, Lines 311-315 in the revised manuscript with track changes)

The sentences have been added as follows: Standard curves for quantifying the concentration of L-(-)-3-PLA in culture supernatants were prepared by dissolving L-(-)-3-PLA (Tokyo Chemical Industry Co., Ltd., Tokyo, Japan) in methanol at concentrations of 1, 5, 10, 50, and 100 μM. Similarly, standard curves for bacterial samples were prepared by dissolving L-(-)-3-PLA in methanol at concentrations of 0.00064, 0.0032, 0.016, 0.08, 0.4, 2, 10, and 50 μM. These samples were also filtered and transferred to HPLC vials.

(Page 20, Lines 341-347 in the revised manuscript with track changes)

The sentences have been added as follows: The concentration of L-(-)-3-PLA in each sample was calculated based on the standard curve. For bacterial samples, the concentration was converted to the amount contained in 50 μL of the bacterial suspension applied to NGM. Specifically, the L-(-)-3-PLA concentration (μM) in the methanol extract, as determined using the standard curve, was used to calculate the amount of L-(-)-3-PLA in the total volume (5 mL) of the bacterial extract. Since 50 μL of bacterial suspension can be obtained 20 times from 100 mL of culture medium, the amount of L-(-)-3-PLA in the 5 mL extract was divided by 20 to calculate the amount of L-(-)-3-PLA in 50 μL of bacterial suspension.

(Pages 32-34, Lines 562-592 in the revised manuscript with track changes)

The sentences have been added as follows:

**The effects of L-(-)-3-PLA on the vulnerability of *P. copri* JCM 13464^T^ and intestinal permeability in *C. elegans***

To evaluate the impact of L-(-)-3-PLA on the vulnerability of *P. copri* JCM 13464^T^ and its effect on intestinal permeability in *C. elegans*, the concentration of L-(-)-3-PLA in the TJ culture supernatant of *P. copri* JCM 13464^T^ and in the bacterial suspension applied to NGM was calculated. The results showed that the concentration of L-(-)-3-PLA in the TJ culture supernatant of *P. copri* JCM 13464^T^ was 5.5 μM, whereas the concentration of L-(-)-3-PLA in the TJ medium-cultured *P. copri* JCM 13464^T^ bacterial suspension applied to NGM was 10 nM (0.5 pmol per 50 μL).

To evaluate the impact of L-(-)-3-PLA on the vulnerability of the bacterial strains, each strain was cultured in BG medium with or without supplementation of 5.5 μM L-(-)-3-PLA. The bacterial survival rate following application to NGM was then assessed. *S. salivarius* JCM 5707^T^ exhibited a significantly smaller change in turbidity in the BG medium supplemented with L-(-)-3-PLA compared to the control group cultured in BG medium alone. No significant differences in turbidity changes between groups were observed for *P. copri* JCM 13464^T^, *M. funiformis* JCM 14723^T^, and *E. coli* (Fig. 6C).

The potential direct impact of L-(-)-3-PLA on intestinal permeability in *C. elegans* was evaluated. *C. elegans* were cultured on NGM plates containing 0, 10, or 100 nM of L-(-)-3-PLA (with *E. coli* OP50 as the food source), and the Smurf assay was conducted. The results showed no significant differences in the proportion of Smurf individuals between any of the three groups (Fig. 8). However, a significant difference in the proportion of Smurf individuals was observed when comparing the control group (0 nM) with the 10 nM group, which corresponds to the concentration found in bacterial samples cultured in TJ medium (*P* = 0.04, Fig. 8, dashed line).

**Fig 8. Effect of L-(-)-3-phenyllactic acid on intestinal permeability in *Caenorhabditis elegans*.** *Escherichia coli* OP50 was cultured overnight in BG medium, concentrated by centrifugation, and applied to nematode growth medium (NGM) supplemented with 0, 10, or 100 nM L-(-)-3-phenyllactic acid (L-(-)-3-PLA). The Smurf assay was conducted as described in Fig 1. The numbers above each bar in the graph indicate the actual count of Smurf individuals among all worms analyzed. The proportions of Smurf individuals were compared between groups using the Holm-adjusted Fisher’s exact probability test. Additionally, a separate comparison between the 0 nM and 10 nM L-(-)-3-PLA groups was performed using the unadjusted Fisher’s exact probability test. *P-*values are shown only for comparisons between groups with significant differences. NS, not significant

(Pages 38-40, Lines 660-695 in the revised manuscript with track changes)

The sentences have been added as follows: Therefore, we added L-(-)-3-PLA at the same concentration (5.5 μM) detected in the TJ culture supernatant of *P. copri* JCM 13464^T^ to BG medium and cultured the bacterial strains. After applying them to NGM, we evaluated their post-application viability. The addition of L-(-)-3-PLA to BG medium inhibited the growth of *S. salivarius* JCM 5707^T^ after NGM application but had no observable impact on the growth or survival of *P. copri* JCM 13464^T^ and *M. funiformis* JCM 14723^T^. In other words, L-(-)-3-PLA alone did not exhibit the same modulatory as TJ medium. Although the resistance mechanism of *Prevotella* to L-(-)-3-PLA has not been elucidated, the unchanged survival of *P. copri* JCM 13464^T^ which produces PLA, under L-(-)-3-PLA supplementation, and the inhibited growth of *S. salivarius* JCM 5707^T^, which does not produce PLA (as described in the section below), suggest that *Prevotella* may possess a specific resistance mechanism against L-(-)-3-PLA. It has been reported that the activity of superoxide dismutase increases in *Staphylococcus aureus* cultured in the presence of PLA [36]. Additionally, when the antioxidant activity gene *soxS* in *E. coli* is activated by genetic modification, the growth of *E. coli* in PLA-supplemented medium is significantly enhanced [37]. Therefore, based on these previous reports and as noted above, one of the mechanisms of the antibacterial action of PLA involves the induction of oxidative stress, and enhancing antioxidant activity could serve as a mechanism of resistance to PLA. In this study, LC-MS/MS data showed a decrease in the levels of 2,3,4,9-Tetrahydro-1H-β-carboline-3-carboxylic acid and Cyclo(phenylalanyl-prolyl) in *P. copri* JCM 13464^T^ cells cultured in TJ medium, and these components have been reported to possess free radical-scavenging ability [38,39]. Therefore, it is plausible that *P*. copri JCM 13464^T^ may utilize these antioxidant components as part of its resistance mechanism to L-(-)-3-PLA. Additionally, culturing in TJ medium might have led to an increase in L-(-)-3-PLA production and a reduction in antioxidant compound levels, which could have contributed to enhanced susceptibility to L-(-)-3-PLA.

It has been reported that PLA intake in mice suppresses intestinal permeability [40]. Additionally, studies on human intestinal epithelial cell lines have shown that D-PLA directly exerts an inhibitory effect on intestinal permeability [41]. In *C. elegans*, although the effect of L-(-)-3-PLA on intestinal permeability has not been reported, *C. elegans* fed 3-PLA exhibited lifespan extension, which is mediated by the activation of SKN-1/AFTS-1 [16]. Since the activation of SKN-1 in *C. elegans* has been suggested to contribute to the maintenance of intestinal barrier function [42], it was hypothesized that L-(-)-3-PLA intake may directly suppress intestinal permeability in *C. elegans*. Therefore, in this study, *C. elegans* was fed NGM supplemented with L-(-)-3-PLA to evaluate its effect on intestinal permeability. The results showed that in the group fed 10 nM L-(-)-3-PLA, which was at the same concentration as the bacterial sample of *P. copri* JCM 13464^T^ cultured in TJ medium, intestinal permeability was significantly suppressed compared to the untreated group. Thus, the mechanism by which intestinal permeability was suppressed in *C. elegans* fed *P. copri* JCM 13464^T^ cultured in TJ medium may involve the enhanced bacterial lysis of *P. copri* JCM 13464^T^, and the direct effect of L-(-)-3-PLA produced by *P. copri* JCM 13464^T^ on enhancing intestinal barrier function.

(Pages 44, Lines 770-773 in the revised manuscript with track changes)

The sentences have been added as follows: In summary, the results of this study suggest that TJ may inhibit intestinal permeability by both rendering *P. copri* JCM 13464^T^ vulnerable and inducing the production of L-(-)-3-PLA, which has intestinal barrier-protective effects, by *P. copri* JCM 13464^T^.

(Pages 51-52, Lines 970-972 in the revised manuscript with track changes)

The sentences have been corrected as follows: S4 Table. Effect of culturing in a BG medium supplemented with tomato juice or L-(-)-3-phenyllactic acid on the growth or survival rate of the tested bacterial strains.

(Figures)

The results of the viability assessment after culturing each test bacterial strain in BG medium containing L-(-)-3-PLA, applying them to NGM, and subsequently resuspending them in BG medium were added to Fig. 6C. The evaluation of the effect of L-(-)-3-PLA on intestinal permeability is presented in Fig. 8.

(Tables)

The results of Fig. 6C have been included in S4 Table.

**Point 4:** Providing information about the statistical methods used to analyze the differences in Smurf ratios and survival rates would increase confidence in the reported outcomes.

**Response:** Thank you very much for your comment. The statistical methods used for each analysis are described in the *Materials and Methods* section and in the respective figure legends. However, we agree that the rationale for choosing specific statistical tests could be more clearly explained. In particular, when comparing continuous variables between groups, it would have been more appropriate to first assess the equality of variances before selecting the statistical method.

Therefore, we have revised the statistical analysis procedures as follows: For comparisons between two groups, an *F*-test was performed to assess the equality of variances. For comparisons among three groups, Bartlett’s test for homogeneity of variances was used. If a tendency toward unequal variances was observed (*P* < 0.1), Welch’s *t*-test was applied for both two- and three-group comparisons. In the case of three-group comparisons, *P*-values were adjusted using the Holm method. If no significant difference in variances was detected (*P* ≥ 0.1), indicating that the assumption of equal variances was met, Student’s *t*-test was used for two-group comparisons, and Dunnett’s test was used for three-group comparisons. We have updated the *Materials and Methods* section accordingly in the revised manuscript.

**Details of corrections in “traced” manuscript:**

(Pages 21, Lines 355-361 in the revised manuscript with track changes)

The sentences have been added as follows: For all other assays, tests for homogeneity of variances were conducted prior to the group comparisons. For two-group comparisons, variance equality was assessed using the *F*-test; for three-group comparisons, the Bartlett test was applied. When a potential violation of the equal variance assumption was detected (*P* < 0.1), Welch's *t*-test was used for both two- or three-group comparisons. In the case of three-group comparisons, *P*-values were adjusted using the Holm method. When the equal variance assumption was considered not violated (*P* ≥ 0.1), Student’s *t*-test was used for two-group comparisons, and Dunnett’s test for three-group comparisons.

(Page 27, Lines 458-461 in the revised manuscript with track changes)

The sentences have been added as follows: The results of the Bartlett test of homogeneity of variances indicated significant differences for both FCS^high^PI^+^ (*P* = 0.03) and FCS^low^PI^−^ (*P* = 0.02). Therefore, a Welch's *t*-test with *P*-value adjustment using the Holm method was used to compare the three groups. For all other data, Dunnett's test was performed (vs. unwashed).

(Page 30, Lines 520-525 in the revised manuscript with track changes)

The sentences have been added as follows: The results of an *F*-test or the Bartlett test of homogeneity of variances indicated significant differences between the BG and TJ groups on day 1 of culture in Fig. 6B (*P* = 0.04), and also between the groups in *Prevotella copri* JCM 13464^T^ in Fig. 6C (*P* = 0.05). Therefore, Welch's *t*-tests were used for comparisons between two groups, and Welch's *t*-tests with *P*-value adjustment using the Holm method were used for comparisons between three groups. For all other data, Student's *t*-tests or Dunnett's tests (vs. BG group) were performed for two- or three-group comparisons, respectively.

(Figures)

In the intergroup comparison of the proportion of FCS^high^PI^+^ cells shown in Fig. 4B, the *P*-values between the unwashed group and the first or second wash groups were modified from 0.01 to 0.0003 and from 0.001 to 0.06, respectively. Similarly, in the intergroup comparison of the proportion of FCS^low^PI^−^ cells, the *P*-values between the unwashed group and the first or second wash groups were modified from 0.002 to 0.00005 and from <0.001 to 0.048, respectively.

//////////////////////////////////////////////////////////////////////////////////////////////////////////////////////////////////

Other major corrections

Following the editorial policy of PLOS ONE, we have omitted descriptions labeled as **'data not shown'** where the data were not critical to the interpretation of the main conclusions.

(Page 12 Line 200, and Page 17 Line 279 in the revised manduscript with track changes)
